# Recent Advances in the Synthesis of Ammonium-Based Rotaxanes

**DOI:** 10.3390/molecules15053709

**Published:** 2010-05-25

**Authors:** Dominic Thibeault, Jean-François Morin

**Affiliations:** Département de Chimie, Centre de Recherche sur les Matériaux Avancés (CERMA), 1045 Ave. de la Médecine, Université Laval, Québec, G1V 0A6, Canada; E-Mail: dominic.thibeault@chm.ulaval.ca (D.T.)

**Keywords:** rotaxane, crown ether, synthesis, end-capping reaction, ring-closing reaction, functional rotaxane

## Abstract

The number of synthetic methods enabling the preparation of ammonium-based rotaxanes has increased very rapidly in the past ten years. The challenge in the synthesis of rotaxanes results from the rather weak interactions between the ammonium-containing rod and the crown ether macrocycle in the pseudorotaxane structure that rely mostly on O·H hydrogen bonds. Indeed, no strong base or polar solvent that could break up H-bonding can be used during the formation of rotaxanes because the two components will separate as two distinct entities. Moreover, most of the reactions have to be performed at room temperature to favor the formation of pseudorotaxane in solution. These non-trivial prerequisites have been taken into account to develop efficient reaction conditions for the preparation of rotaxanes and those are described in detail along this review.

## 1. Introduction

**Figure 1:**
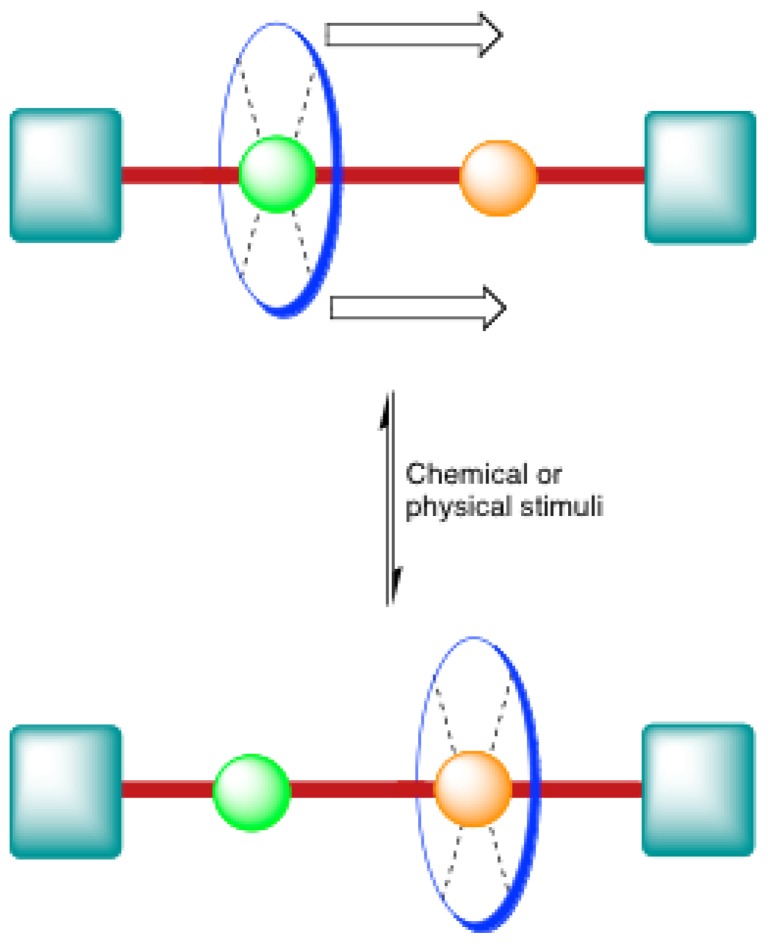
Cartoon representation of a translational motion allowed in a [2]rotaxane.

**Figure 2:**
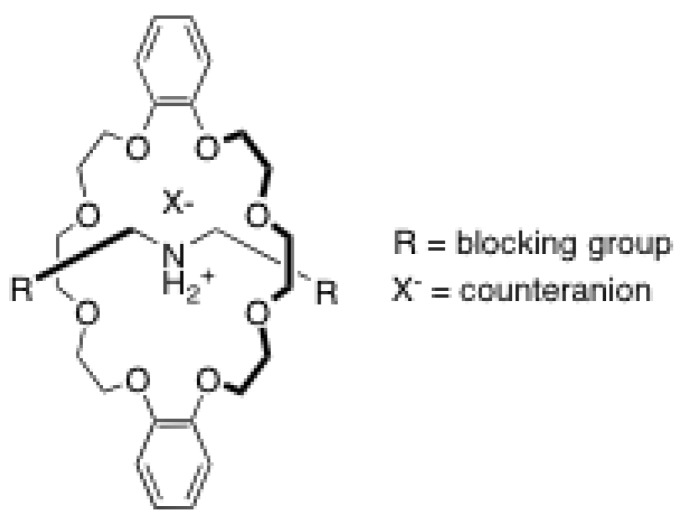
Ammonium-crown ether-based rotaxane.

The rotaxane architecture is considered as one of the most useful tools in the chemist’s toolbox to prepare functional synthetic nanomachines. The interest in these supramolecular architectures comes from their interlocked structure that provides them with high degree of freedom, thus allowing tunable translational motion at the nanoscale when proper chemical or physical stimuli are used (Figure 1). In the past twenty years, a wide variety of rotaxanes with different rods and macrocycles have been developed for different applications. In most cases, rotaxane assemblies rely on hydrogen bonding to ensure thermodynamic stability. Thus, particular efforts have been devoted on the development of rods and macrocycles allowing multiple H-bonds with one to each other. One of the most important types of rotaxane of this class is that having an ammonium-binding site onto the rod encircled by a crown ether (Figure 2). However, this type of rotaxanes is not easily accessible since several reactions conditions must be avoided in their synthesis. Firstly, no polar, protic solvent can be used for the reaction since the formation of the pseudorotaxane, which is the reaction intermediate prior to the formation of the desired rotaxane, relies on H-bonding for thermodynamic stabilization. Secondly, because the secondary ammonium moiety in the rod is acidic, no strong base can be used. Finally, because ammonium-based rotaxanes are thermodynamically stabilized supramolecular architectures, the temperature has to be kept as low as possible during the course of reaction in order to drive the equilibrium toward the formation of the desired pseudorotaxane. Thus, synthetic methods to obtain those ammonium-based rotaxanes in high yield have been developed and research is ongoing to find efficient ways to introduce functional groups directly onto the rotaxane scaffold, leading to functional materials.

In this review, we present the advances in the synthesis and functionalization of ammonium-based rotaxanes over the last ten years. This review is focused mainly on the new synthetic methodologies for efficient preparation of rotaxanes and it is divided in three parts; the synthesis of rotaxanes through end-capping reactions, synthesis through macrocycle ring-closing reactions (clipping procedure) and the synthesis of functional rotaxanes.

## 2. Rotaxane Formation through End-Capping Reactions

### 2.1. Esterification reactions

Among the methods developed in the past years to form rotaxanes in high yield, the strategy involving an end-capping reaction is probably the one that has attracted the most attention. Several efficient reactions have thus been used for this purpose and what follows is an overview of what has been done by many groups in the past ten years.

**Scheme 1:**
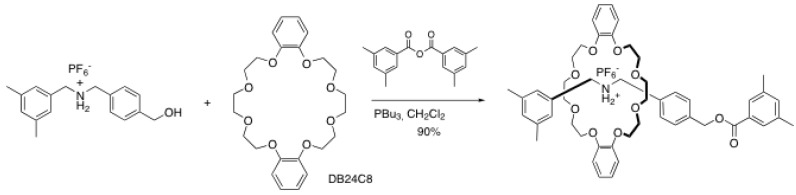
Formation of a rotaxane by an esterification reaction.

The reactions involving a nucleophile at one end of the rod and a bulky molecule having an electrophilic character are by far the most popular way to end-cap a rotaxane. Among others, ester-forming reactions are widely used for this purpose. Takata *et al.* reported one of the first examples by using a hydroxyl-terminated rod and an aromatic anhydride (Scheme 1) [1].

This reaction does not proceed without catalyst, whereas yields up to 90% can be obtained when Bu_3_P is used. Other stronger bases such as DMAP lead to only traces amount of the final rotaxane. The reaction can be accomplished with good to excellent yields in dichloromethane, benzene, dioxane and acetonitrile. Surprisingly, only trace amounts of the desired rotaxane are recovered when an acyl chloride is used as catalyst rather than the anhydride. The authors hypothesized that the leaving group (Cl^-^) acts as a base that is strong enough to deprotonate the ammonium group. However, counteranion exchange resulting in ammonium salts with low solubility can also explain the poor yields obtained. The scope of this reaction has been extended to different components (crown ether and blocking groups) with success. This end-capping reaction has been exploited several times by this group [2,3,4,5,6] and others [7,8,9] to prepare various rotaxane assemblies.

Other acylating reagents have been studied to replace anhydrides, which can be incompatible with some functional groups. One interesting surrogate is the active 2-pyridylthio ester that shows good reactivity with primary alcohols in the presence of Bu_3_P to form a [2]rotaxane in 85% yield [10]. Functional active esters have also been used in the same report to prepare functional [2]- and [3]rotaxanes in good yield. 

Tokunaga *et al*. also performed esterification reactions in which the electrophile is placed at the end of the rod [11]. Their strategy relies on the use of a bulky diaryldiazoalkane to form the ester in the presence of a carboxylic acid group at the end of the rod. Here again, very high yields (up to 97%) can be obtained. The great advantages of this methodology are the mild conditions used and the functional group compatibility.

**Scheme 2:**
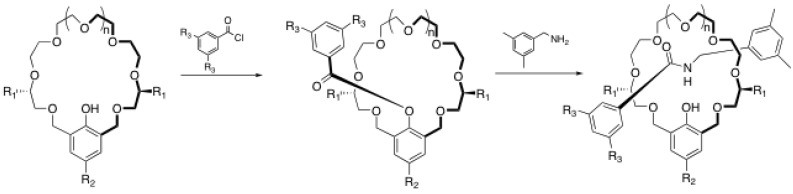
Formation of rotaxane via aminolysis of a prerotaxane.

Hirose *et al*. have recently developed another interesting approach for the synthesis of a rotaxanes using an esterification reaction [12]. They showed that a rotaxane can be formed by the aminolysis of a prerotaxane, which is composed of a phenolic pseudo-crown ether as the ring and a bulky stopper (Scheme 2). The yield for this reaction is very good (82% under optimized conditions) and more surprisingly, the selectivity of rotaxane over the formation of the dumbbell reach 100%. The selectivity is better when non-polar solvents are used (C_6_D_6_ for example) rather than polar solvents such as DMF. This result shows that the crown ether plays an essential role in approaching the amine group from the backside, allowing the formation of the rotaxane rather than the dumbbell, as shown in Scheme 2.

**Scheme 3:**
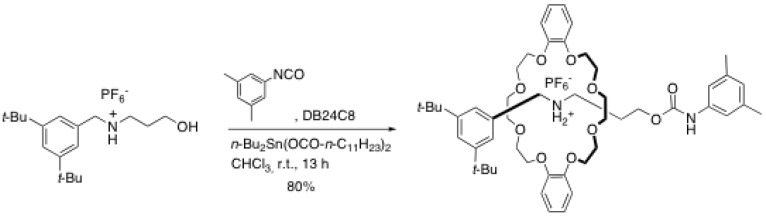
Formation of rotaxane via urethane formation.

Although ester formation has proven to be particularly useful for the formation of rotaxanes, this functional group suffers from relatively poor stability towards nucleophiles and electrophiles as well as reducing agent and some oxidizing agents. Thus, the preparation of rotaxanes through the formation of a more robust functional group was desirable. In this context, Takata *et al.* developed a strategy in which an urethane group is created from an hydroxyl-containing rod and a bulky isocyanate derivative (Scheme 3) [13]. Urethanes are known to be more stable than ester linkages towards aggressive reagents. The best yields (~90%) were obtained by using 0.1 equivalent of dibutyltin dilaurate as catalyst and two equivalents of DB24C8 in non polar solvents such as chloroform, chlorobenzene or nitromethane at room temperature. As for other rotaxane formation reaction, no rotaxane was recovered when a polar solvent (DMAc) was used. Because of its high efficiency and the commercial availability of 3,5-dimethylbenzene isocyanate, this method has become very popular and several research groups involved in the rotaxane synthesis have employed this method routinely [14,15,16,17,18,19].

### 2.2. Reversible disulfide and imine formation

**Scheme 4:**
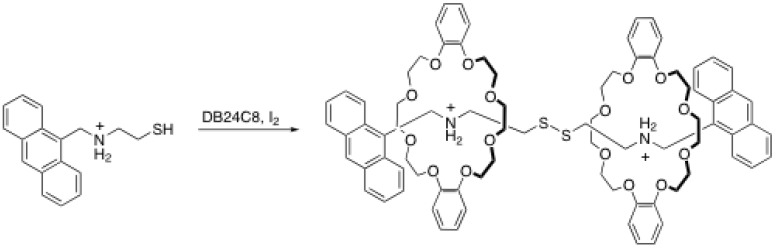
Formation of rotaxane via disulfide formation.

**Scheme 5:**
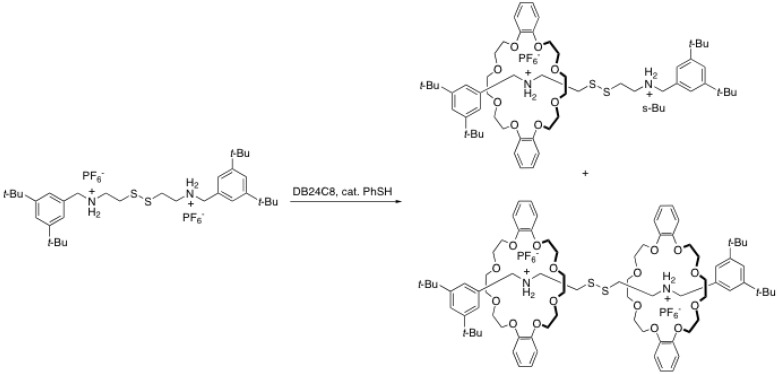
Equilibrium process during the formation of [3]rotaxane through disulfide formation.

Busch *et al.* proposed in the late 90’s a strategy to prepare a reversible rotaxane (rotaxane/ pseudorotaxane equilibrium) [20]. Their strategy consists in the formation of a disulfide by oxidizing a pseudorotaxane bearing a thiol (-SH) on the rod with iodine (Scheme 4). This leads in very good yield (84%) to symmetrical [3]rotaxanes, in which the two crown ethers lie close one to each other [20]. The most interesting feature of the disulfide formation is the reversibility of that bond. Indeed, simply by mixing a blocked rod containing two ammonium salts, a crown ether and a catalytic amount of benzenethiol one can form rotaxanes [21]. Because the disulfide bond is reversible, the rod will be separated into the thiol derivatives after reaction with benzenethiol, allowing them to insert the crown ether to form a pseudo[2]rotaxane. The disulfide bond is formed again to provide the rotaxane. This process is presented in Scheme 5.

An important drawback of this reaction is the time needed to form the [3]rotaxane in good yield. The best results have been obtained at room temperature for a reaction time ranging from ten to thirty days. Below that time, most of the material recovered is the [2]rotaxane [22,23]. This strategy has also been used to prepare low molecular weight poly(rotaxane)s [24].

Similarly, a rotaxane can be formed in a reversible fashion by using an imine bond formation between a rod bearing aldehyde and a bulky amine stopper [25]. An interesting feature of the imine compared to disulfide is that the imine can be reduced to provide a kinetically inert rotaxane. Moreover, the formation of an imine followed by its reduction and protonation can provide an additional ammonium site for the crown ether macrocycle [26].

### 2.3. Wittig reaction

**Scheme 6:**
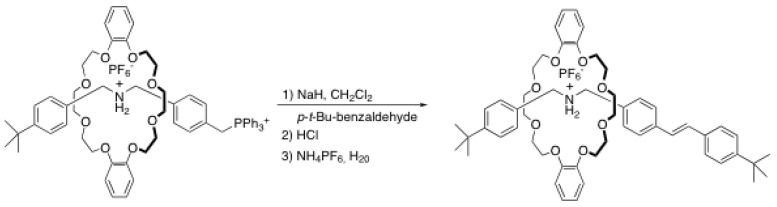
Rotaxane formation through Wittig reaction.

A very attractive strategy to prepare rotaxanes using the capping methodology has been developed in the early 2000s. Thus, Stoddart *et al.* used the Wittig reaction between a pre-rotaxane bearing a triphenylphosphonium stopper and a bulky moiety bearing aldehyde [27,28,29] (Scheme 6).

The first step of this synthesis was to form the pre-rotaxane **1**-H•2PF_6_ from the bromobenzyl derivative of the rod, DB24C8 and PPh_3_ [27,29]. Then, **1**-H•2PF_6_ was treated with NaH as a base followed by a bulky benzaldehyde derivative. The yields usually obtained for these rotaxanes are high considering the fact that the NH_2_^+^ recognition site can be deactivated by the use of a strong base. The alkene then formed can be reduced to a simple alkane with PtO_2_ and hydrogen [28]. One of the most interesting features of this reaction is that the rotaxane rod can be elongated and that a second ammonium station can be added without adding an additional crown ether. This method is thus particularly useful in the context of the use of rotaxane as nanomachines in which a crown ether macrocycle can switch from one station to another under the influence of a stimulus (Figure 1). A few months after the initial report of the Wittig strategy for rotaxane preparation, Stoddart *et al*. prepared a series of cyclic and acyclic “daisy chain” polymers from a [2]rotaxane with a crown ether bearing an aldehyde functional group and a triphenylphosphonium stopper [30]. In the years that followed Stoddart *et al*. published several articles involving this strategy for the synthesis of oligoethylene glycol-based rotaxanes [31], [3]catenanes and [3]rotaxanes [32] and dendrimers with rotaxane-like branching [33]. The major drawback of this strategy is the use of a strong base, which is incompatible with several kinds of functional groups. This decreases significantly the scope of this reaction and other methods involving an end-cap exchange strategy have been developed to address this (see below).

### 2.4. Metal-catalyzed reactions

**Scheme 7:**
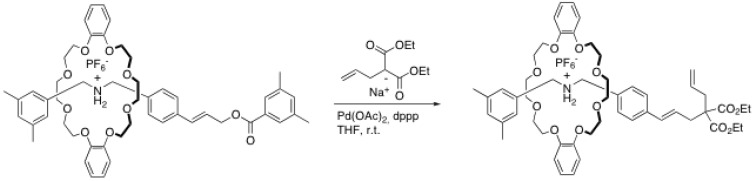
End-cap exchange through Tsuji-Trost allylation reaction.

With the aim developing new versatile tools for the synthesis of rotaxanes under mild conditions and to obtain functional complex rotaxanes, many research groups have put their attention on metal-catalyzed reactions for the capping step. Takata’s research group has been particularly active in the development of such capping reactions. In 2005, they reported the synthesis of a [2]rotaxane through a Tsuji-Trost allylation reaction, as shown in Scheme 7 [34]. 

The strategy employed is very similar to that used by Stoddart *et al.* employing the triphenylphosphonium group. First, a pre-rotaxane is formed using an end-cap reaction through ester formation [1]. Then, a Tsuji-Trost allylation is performed with sodium diethylallylmalonate, bearing an acid-sensitive allyl group and base-sensitive ester groups. After optimization of the reaction conditions, it was found that 5 mol% of Pd(OAc)_2_ as catalyst and 10 mol% of dppp as ligand in THF gives the best yield (96%) [34]. It is noteworthy that the success of this reaction depends strongly on the steric hindrance near the alkene on the rod. Indeed, if the crown ether is too close to the alkene, the allylation the yields were found to be low. Thus, longer rods have to be used to ensure the success of this reaction. 

**Scheme 8:**
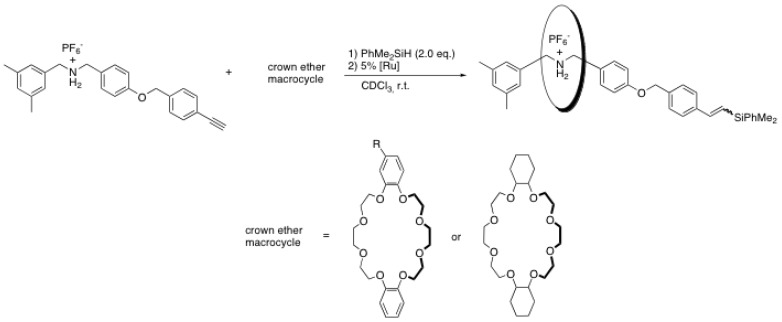
Rotaxane formation through metal-catalyzed hydrosilylation reaction.

Similarly, the same group synthesized both simple and complex rotaxanes using the transition metal-catalyzed hydrosilylation of alkynes as the blocking reaction (Scheme 8) [35]. Under the optimized conditions, yields up to 88% for the [2]rotaxane can be obtained with excellent regio- and stereoselectivity. Among others, Wilkinson’s catalyst RhCl(PPh_3_)_3_ and RuHCl(CO)(PPh_3_)_3_ were found to be the most effective catalysts for this reaction. This approach is particularly interesting since the alkyne-containing rod can be easily obtained through Sonogashira coupling and the hydrosilylation reaction proceeds at room temperature in non-polar solvents. Moreover, several silane derivatives containing bulky substituents such as phenyl and branched alkyl are commercially available.

**Scheme 9:**
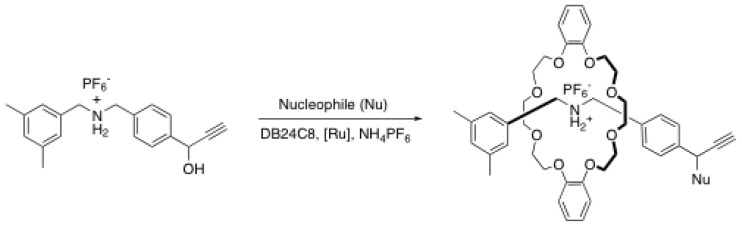
Rotaxane formation through ruthenium-catalyzed propargylic substitution.

Tokunaga *et al*. have developed another metal-catalyzed end-capping reaction involving an alkyne-containing rod, namely a ruthenium-catalyzed propargylic substitution [36]. The greatest advantage of this method is the possibility of introducing various functional nucleophilic blockers (Scheme 9). 

Indeed, the substitution can be achieved through nitrogen, sulfur, carbon and phosphorus nucleophiles. Under the optimized conditions, yields up to 97% of the rotaxane can be obtained, even if the reaction mixture has to be heated at 60 ºC to proceed. Because the alkyne remains intact during the course of the reaction, one can think of rotaxane post-functionalization reaction to introduce functional groups to provide the rotaxane with specific properties for different applications. 

**Scheme 10:**
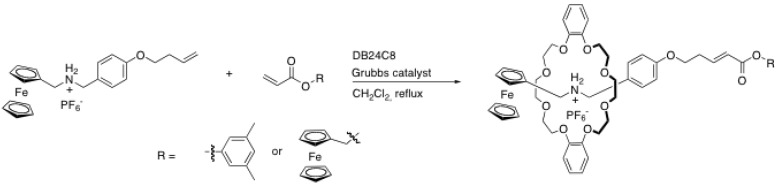
Rotaxane formation through ruthenium-catalyzed cross-metathesis.

Ruthenium-catalyzed cross-metathesis reaction has also been exploited to end-cap ammonium-crown ether-based rotaxanes. This reaction possesses several advantages for rotaxane formation: it proceeds well at room temperature, it does not require base, it works well in non-polar solvents and the catalyst has a high tolerance to several functional groups [37]. Osakada *et al*. have been the first to report such a strategy to end-cap a rotaxane with a ferrocenyl moiety on one end and an olefin at the other end (Scheme 10) [38]. While the vinylbenzene-containing rod does not react with 3,5-dimethyl-phenyl acrylate using the second generation Grubbs catalyst due to steric congestion around the crown ether macrocycle, the vinylalkyl-containing rod reacted in 72% yield. The same group has used this reaction to prepare a ferrocene-based electroactive [3]rotaxane in 50% yield [39].

**Scheme 11:**
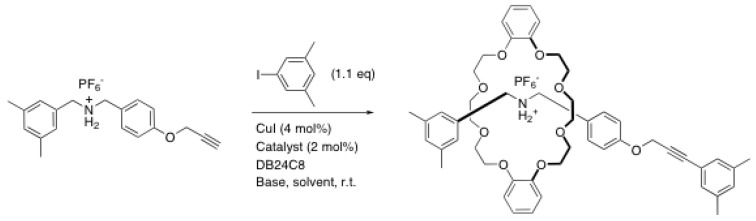
Rotaxane formation through Sonogashira coupling.

Recently, Morin et al. have used the Sonogashira coupling between an alkyne-containing rod and a bulky iodoarene derivative to end-cap rotaxanes (Scheme 11) [40]. The most important drawback of this reaction is the necessity to use a base to form the alkynecuprate intermediate. Thus, a variety of different bases has been tested in the Sonogashira coupling and the best results were obtained with triethylamine and *N*-isopropyl-*N*-methyl-*tert*-butylamine. Even in the best cases however, the yield was only 26% for the rotaxane formation. This can be attributed to the reaction conditions that are not the best for the Sonogashira coupling such as the solvent (acetonitrile was used) and the low amount of base used (1.0 equivalent). Nevertheless, a [3]rotaxane was successfully synthesized using this methodology [40].

### 2.5. Cycloaddition reactions

**Scheme 12:**
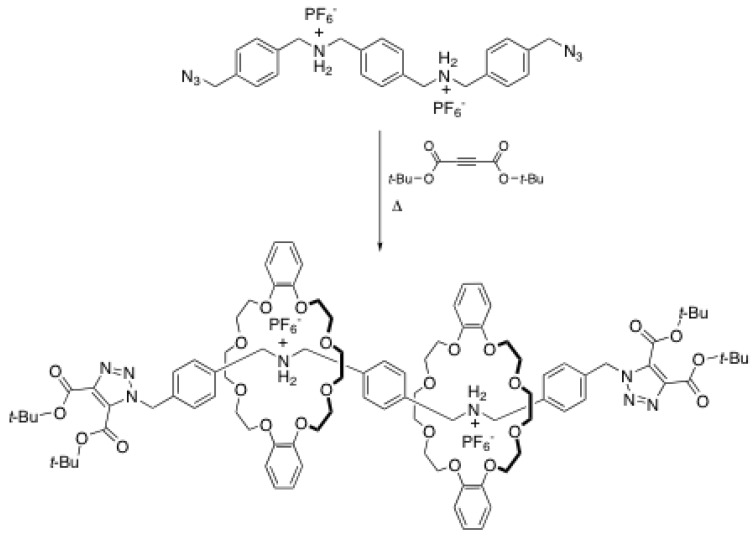
Rotaxane formation through 1,3-dipolarcycloaddition.

Although used to a lesser extent than the reactions described above, cycloaddition reactions have proved to be very useful for the end-capping of ammonium-based rotaxanes. When a proper set of reactants is used, cycloaddition reactions can proceed without additives, thus facilitating the purification step. Stoddart *et al*. reported one of the first examples of a cycloaddition end-capping reaction in the early 2000s [41]. They reported the synthesis of a [3]rotaxane through a bicycloaddition reaction between a rod with azides at both ends and bulky acetylenedicarboxylate acting as a electron-deficient dipolarophile (Scheme 12). The reaction proceeds well in dichloromethane, but the solution has to be heated to reflux for four days to obtain a 77% yield. Interestingly, when only one equivalent of DB24C8 is used to perform the synthesis of the [3]rotaxane, only the starting rod and the desired product were isolated without any trace of the one-station [2]rotaxane. This has been attributed to the poor solubility of the [2]pseudorotaxane in dichloromethane.

Recently, Coutrot *et al*. used the copper(I)-catalyzed Huisgen alkyne-azide 1,3-dipolar cyclo-addition to end-cap a [2]rotaxane using an azide-containing mannopyranose blocking group [42,43]. The advantage of this reaction is the possibility to perform the reaction at room temperature, in opposition to the mild heating needed for the same reaction without copper (I) catalyst [41]. As a base, the authors used 0.1 equivalent of 2,6-lutidine, which is bulky enough to not deprotonate the ammonium before and after rotaxane formation. Cu(MeCN)_4_PF_6 _has been used as copper (I) catalyst to presumably avoid anion exchange during the course of the reaction. With all this careful attention to the reaction conditions, a [2]rotaxane has been prepared in 75% yield. The same group used this reaction to prepare a new switchable mannosyl-based daisy chain molecular machine in an excellent yield of 92% [44]. This particular molecule of higher degree of freedom could be of particular interest in biological events in which multivalent interaction is desired for ligand/receptor recognition. Using the same click strategy, Coutrot *et al*. were also the first to explore the possibility of creating an efficient second station for the DB24C8 by alkylation of the triazole, with the aim of synthesizing molecular machines [43].

**Scheme 13:**
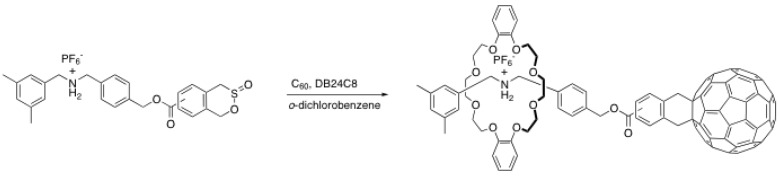
Rotaxane end-capping with C_60_ through Diels-Alder reaction.

Cycloaddition reactions have also been used to end-cap rotaxanes with C_60_. Because C_60_ is known to be a relatively good dienophile, a rod containing a diene or its precursor can be used to achieve a Diels-Alder reaction with it. Takata *et al*. have demonstrated this concept using a sultine derivative, which, upon heating in *o*-dichlorobenzene, forms a reactive diene for a Diels-Alder reaction with C_60_ (Scheme 13) [45]. Because of the relatively high temperature needed to perform this reaction (80 ºC), the reaction yield is quite low at 33%. However, the varieties of reactions that can be used to attach pristine C_60 _covalently to other moieties are rather limited and often involve heating. Nevertheless, this method is interesting since it can be used to prepare functional, complex rotaxane architectures with unique properties.

### 2.6. Solvent-free reactions

For all the methods discussed so far to prepare rotaxanes, the yields of formation are limited by different factors, but mostly by the association/dissociation equilibrium between the crown ether and the ammonium rod. This equilibrium depends intrinsically on the association constant between the macrocycle and the rod and thus on the solvent used to perform the complexation. In the best-case scenario, pseudorotaxane could be formed under solvent-free conditions, thus eliminating the dethreading of the pseudorotaxane once formed. However, an efficient reaction in solvent-free conditions has to be found and exploited to end-cap rotaxanes. Chiu *et al*. have successfully addressed this challenge in 2008 by synthesizing a [2]rotaxane in the solid-state through the formation of a hexahydropyrimidine by condensing an aldehyde-containing rod with 1,8-diaminonaphthalene as the bulky stopper [46]. In a first step, the ammonium rod and the macrocycle were mixed in acetonitrile and the solvent was removed under reduced pressure to provide a white powder containing predominantly the pseudorotaxane. Then, this powder and 1,8-diaminonaphtalene were ball-milled together at room temperature for one hour. After usual column chromatography, the [2]rotaxane was isolated in 80% yield (87% if the amount of macrocycle is increased). This method is so efficient that a [4]rotaxane can be synthesized in 78% yield starting from a triangular-shape rod.

The same group has extended the scope of this technique by using an efficient Diels-Alder reaction between 1,2,4,5-tetrazine and a terminal alkyne to prepare the smallest rotaxane ever reported in the literature [47]. Using a similar synthetic procedure, a simple [2]rotaxane has been prepared in 81%. Various tetrazine derivatives have been used to end-cap rotaxanes in good yield and different macrocycles have been successfully used [48]. 

### 2.7. Miscellaneous reactions

Because of the great interest toward rotaxanes as functional materials and nanomachines in the past ten years, various blocking groups with specific properties and functions have been used to end-cap rotaxanes. For obvious synthetic reasons, those functional blocking groups can be introduced using particular reactions other than those presented in this review so far. For example, Asakawa *et al.* proposed to introduce a metalloporphyrin into the structure of a rotaxane by using it as a blocking group [49]. The blocking reaction takes place thanks to the strong interactions between a pyridine-containing rod and a Rh-porphyrin in 70% yield. In order to obtain biologically active rotaxanes, Coutrot *et al.* prepared a glycorotaxane through an *o*-glycosylation of an anilinium derivative in the presence of DB24C8 [50]. This method is an efficient access to rotaxanes (up to 91% in less than 1 minute), with a high selectivity toward mannosylorthoesterrotaxane (74%) rather than *O*-mannosyl-dicrotaxanes (17%). Conversion of orthoesterrotaxanes to *O*-mannosyldicrotaxanes is more tricky (49%) as a result of a side deacetylation of the C2 hydroxyl of the glucidic skeleton and side dethreading [50]. Also, this method often leads to a mixture of different isomers and the isolated yield of rotaxane was rather low.

Lots of other known organic transformations have also been applied in the synthesis of rotaxanes in order to develop a better, “universal” method to end-cap them. Among others, blocking reactions involving 1,3-dicyclohexylcarbodiimide (DCC) [51], trityl [52], diethylphosphoramidate [53], diphenylmethane [54], platinum complex [55], cycloheptadiene [56], substituted maleimide [57], boroxine [58] and pyridinium [59] have been used successfully to end-cap rotaxanes with various success. 

## 3. Rotaxane Formation through Macrocycle Ring-Closing Reactions

**Scheme 14:**
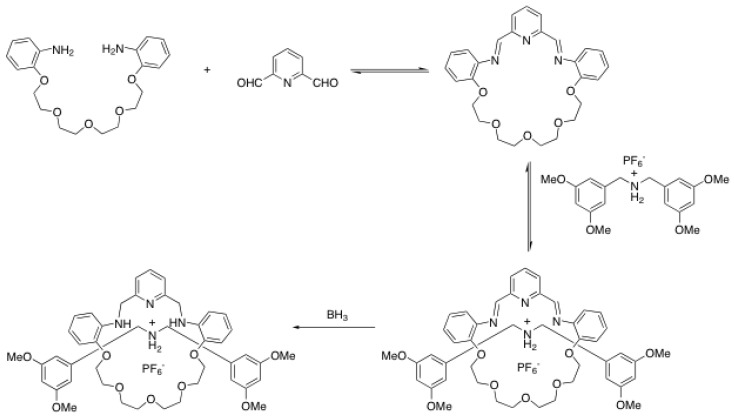
Rotaxane formation through clipping of the macrocycle.

Although the macrocycle ring-closing reaction, also known as the “clipping protocol”, has been less employed than the traditional end-cap reactions to prepare rotaxanes, very interesting and efficient methods have been reported in the last ten years. Stoddart *et al*. were the first to propose such a strategy to build rotaxanes under thermodynamic control [60]. As the clipping reaction, they used the condensation between 2,6-pyridinedicarboxaldehyde and tetraethyleneglycol bis(2-aminophenyl)ether (Scheme 14) [60]. In the two step condensation-reduction process, they obtained the desired [2]rotaxane in 70% yield after standard column chromatography. Stoddart *et al*. used this strategy later on to assemble mechanically interlocked dendrimers [61], long rotaxaneoligomers [62] and to study the dynamics of reversible rotaxanes through imine bond formation [63].

**Scheme 15:**
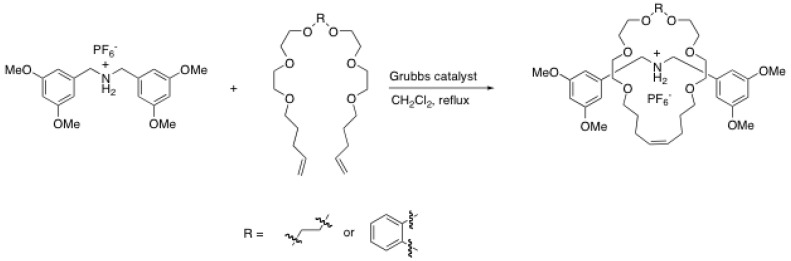
Magic ring rotaxanes synthesis by ruthenium-catalyzed olefin metathesis.

Soon after the first report of Stoddart *et al*., Grubbs *et al*. proposed to use the ruthenium-catalyzed olefin metathesis to perform the ring closure reaction of a crown ether macrocycle over a dumbbell-shaped template containing an ammonium ion [64]. The use of ruthenium metathesis for the synthesis of rotaxane is particularly useful since it is done in mild conditions in non-polar solvents under gentle reflux in dichloromethane without base. Using the first generation catalyst, the [2]rotaxane has been obtained in 73%. The yield of the reaction depends strongly on the chemical nature of the crown ether. In fact, replacing the -CH_2_CH_2_- group by an *ortho-*substituted phenyl ring leads to a dramatic decrease of the reaction yield (73 to 30%) (Scheme 15). 

Asakawa *et al.* used this strategy to introduce functional groups inside the macrocycle of the rotaxane with the aim to complex metal ions inside rotaxanes to change their size and shape [65]. Hence, by introducing hydroxyl group, they have been able to complex palladium(II) [65] and nickel(II) [66]. Finally, Osakada *et al*. used the ruthenium-catalyzed olefin metathesis to synthesize rotaxane with more complex macrocycle having specific functions [67].

## 4. Functionalization of Ammonium Ion in Rotaxanes

The modification of rotaxanes is an interesting way to add functional groups onto the rotaxane structure. One strategy that can be used is to deprotonate the ammonium group of the rod and to modify it with different electrophiles. Takata *et al*. studied extensively this reaction in the early 2000s after observing that an ammonium group encircled by a crown ether in the rotaxane configuration has very low acidity compared to the same ammonium free of crown ether [68]. In this study, they optimized the reaction conditions and found that two equivalents of acyl chloride in the presence of five equivalents of triethylamine in acetonitrile gave the best results with nearly 100% conversion in the best cases. Using these conditions, acetyl and benzoyl can be added onto the ammonium group very efficiently. This group used these conditions in many subsequent reports to synthesize different rotaxane assemblies [2,3,4,34,69,70]. In addition to acylation reaction, methylation can be performed on the ammonium unit using formaldehyde and formic acid in DMF in quantitative yield [3].

## 5. Synthesis of Functional Rotaxanes

Beside the synthetic aspect, rotaxane is a very interesting and promising supramolecular architecture for many applications. However, because of its interlocked nature, rotaxanes have been synthesized mostly as a nanomachine component in which a translational motion can be induced by various stimuli [71]. In order to respond to such stimuli and to accomplish complex tasks at the nanoscale, rotaxanes have to be modified with proper functional moieties, including photo-, electro- and bioactive units [72]. Hence, different strategies to attach such units on rotaxane scaffold in order to influence their properties have been developed. Here is a brief summary of the different approaches developed in the past years to functionalize rotaxanes for different applications. 

### 5.1. Bioactive rotaxanes

Two research groups are actively involved in this research area. The Coutrot group reported the synthesis of rotaxanes containing glycosides to study the high flexibility that rotaxane architectures offer for multivalent recognition [42,43,44,50]. In all the examples they reported so far, the functional moiety (glycoside) act as the stopper for the rotaxane. Using the rotaxane architecture and its interlocked nature, they have been able to tailor the distance between different mannosyl groups. 

In order to also exploit the flexibility offered by the rotaxane scaffolds, Smithrud *et al*. have prepared a large variety of rotaxanes with amino acids attached directly to the macrocycle. Some examples of such rotaxanes are shown in Figure 2. In their model, the ring motion is used as a mimic of the peptide loops that cover protein binding sites [73]. Thus, they synthesized multiple crown ethers with different pending groups, including amino acids, and studied their ability to act as artificial receptors. Structure-properties relationship has also been studied in regard to the binding ability [74]. These so-called host-[2]rotaxanes have been tested as cellular transport agent for fluorescein-PKC inhibitor conjugate [75,76,77,78].

### 5.2. Photo- and electroactive rotaxanes

Because development of nanomachinery is the first motivation behind the preparation of various types of rotaxanes, complex structures with photo- or electroactive moieties that can produce useful mechanical work upon light or electrochemical stimulation have been prepared over the years. What follow are representative examples of what have been done in this area with ammonium-based rotaxanes. 

**Figure 3:**
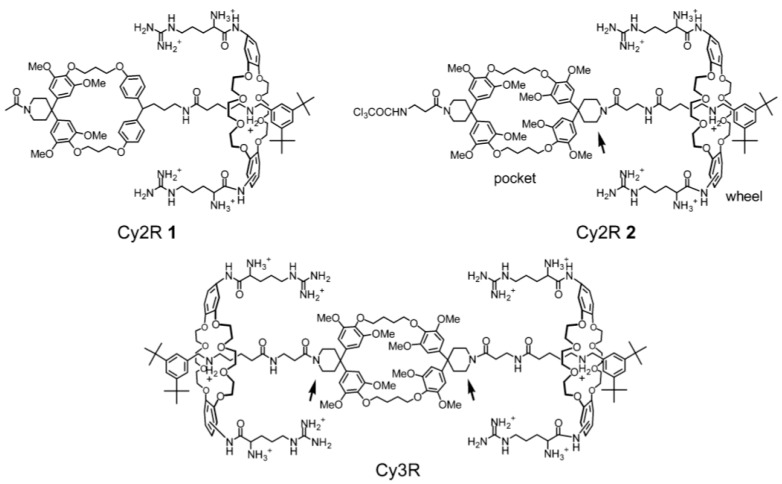
Examples of rotaxane bearing amino acids moieties.

**Scheme 16:**
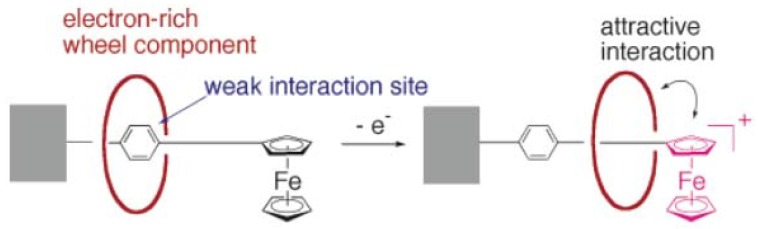
Electrochemically-induced translational motion in rotaxane.

Ferrocenes appear as an important electroactive unit in the preparation of rotaxanes since they can be introduced into a rotaxane structure using various types of reaction. Moreover, ferrocenes are bulky enough to mechanically lock the rotaxane. Takata *et al*. exploited these properties to synthesize a simple rotaxane blocked with a ferrocene unit using an esterification reaction (Scheme 16) [79]. When the rotaxane is in its neutral form, the crown ether macrocycle encircled the phenyl moiety, which is rather weak interaction site. Upon oxidation of the ferrocene unit, the macrocycle move toward the redox center where stronger interaction can take place. The macrocycle can return to its original position after reduction of the ferrocene moiety.

Later on, the same group published the synthesis and photophysical study of a rotaxane with both ferrocene and fullerene in their structures [80,81]. In those cases, the rotaxane structure acts as a template to create close contact between ferrocene, an electron-donating unit, and the fullerene, an electron-accepting unit in order to study potential through-space electronic interactions between both units. Similar studies have been performed on a rotaxane scaffold having a tetrathiafulvalene (TTF) [82] or an OPV unit [83]. Those complexes open the way to the preparation of efficient artificial photosynthetic systems.

Finally, Morin *et al*. used the rotaxane architecture as a scaffold for fullerene complexation (Figure 3) [18]. By functionalizing the two crown ether macrocycles of a [3]rotaxane with a porphyrin unit, they have been able to capture fullerenes of various sizes and shape with association constant ranging from 10^3^ to 10^5^ M^-1^. Analogues of this rotaxane have been fixed to a gold surface in order to immobilize pristine C_60_ on surface [19]. However, the self-assembled monolayers formed from these rotaxanes are not well organized and the complexation study with C_60_ has not been successful.

**Figure 4:**
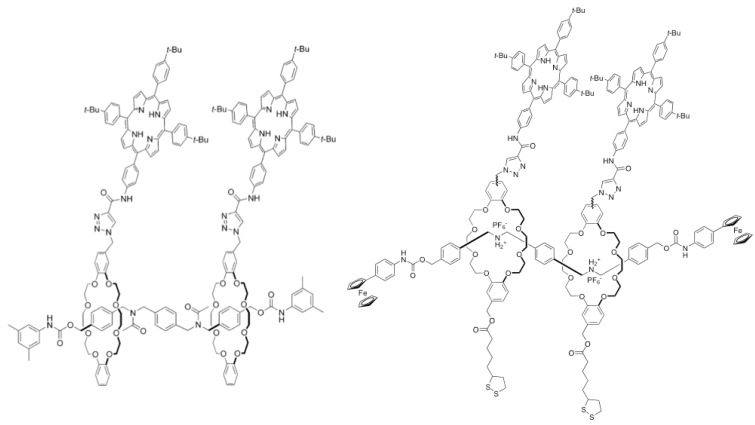
Supramolecular host for fullerenes based on rotaxane scaffold.

## 6. Conclusions

As demonstrated in this review, the number of synthetic methods available to chemists to efficiently prepare ammonium-based rotaxanes has increased very rapidly and one can argue that chemists and material scientists have now plenty of available strategies to prepare novel, very complex rotaxane architectures for different applications. This has been exemplified in the last three or four years with the preparation of very intricate, multifunctional rotaxanes for purposes ranging from biomedical to electronic applications. Nonetheless, the use of rotaxanes as functional materials is still at its early stage and it is obvious that the high degree of freedom along with the mechanically interlocked nature of rotaxanes have been underexploited. One can imagine that with all the new and quite easy synthetic methods developed recently, ammonium-based rotaxanes will become one of the most widely used scaffolds for a broadening range of applications.
